# COVID anxiety and its predictors among Slovak adolescents

**DOI:** 10.3389/fpsyg.2022.993003

**Published:** 2022-12-12

**Authors:** Bibiána Jozefiakova, Natália Kascakova, Jana Furstova, Gabriela Sarnikova, Jozef Hasto, Peter Tavel

**Affiliations:** ^1^Olomouc University Social Health Institute, Palacky University, Olomouc, Czechia; ^2^Psychiatric-Psychotherapeutic Outpatient Clinic, Pro Mente Sana, Bratislava, Slovakia; ^3^Department of Christian Education, Sts. Cyril and Methodius Faculty of Theology, Palacky University, Olomouc, Czechia; ^4^Department of Social Work, St. Elizabeth College of Health and Social Work, Bratislava, Slovakia

**Keywords:** COVID-19, adolescents, general health, COVID anxiety, attachment, resilience

## Abstract

**Introduction:**

The COVID-19 pandemic and its related restrictions, mainly social distancing, had an impact on the mental health of various groups, including adolescents.

**Methods:**

The main goal of our study was to explore the impact of gender, age, resilience (measured using the Brief Resilience Scale), attachment anxiety, attachment avoidance (both measured using the Experiences in Close Relationships Revised Scale for adolescents), and mental and general health (measured using items of SF-8 Health Survey) on COVID anxiety (measured using the COVID Anxiety Scale) among a sample of Slovak adolescents (*N* = 1,786, age 15 to 19, mean age = 16.8, SD = 1.2). The data were collected online between 13 April and 24 May 2021.

**Results:**

Four nested linear regression models were fitted to the data and evaluated. The significant predictors that had a greater effect than our smallest effect size of interest (*β*  = 0.10) were gender (*β*  = −0.26, *p*  < 0.001, where boys had lower scores in COVID anxiety), general and mental health (*β*  = −0.13 and *β*  = −0.14, respectively, both with *p*  < 0.001), resilience (*β*  = −0.12, *p*  < 0.001), and attachment avoidance (*β*  = −0.11, *p*  < 0.001). Similarly, age and attachment anxiety were significant predictors with a lower effect size (*β*  = 0.06, *p*  = 0.003, and *β*  = 0.09, *p*  < 0.001, respectively).

**Discussion:**

Our results are in line with previous research findings highlighting the importance of prevention and interventions programs focused mainly on preventing loneliness and social disconnection, fostering secure attachment with parents and peers, and increasing the resilience of adolescents, especially in the stressful time of a pandemic, to promote their mental health.

## Introduction

Over the last 2 years, the COVID-19 pandemic has significantly affected the lives of all people, including adolescents. The restrictions associated with the pandemic, which mainly involved social distancing, had a great impact on the functioning of the school system. In many countries, including Slovakia, students in primary, secondary and higher education shifted, after sudden breaks, completely to distance learning, without the possibility of meeting their classmates and teachers. Besides education, young people also experienced other negative consequences of the pandemic, such as the financial and job instability of their families, the disruption of their general and family social relationships, acute and even chronic stress, and more.

In general, stress and stressful life events are significant predictors of mental health difficulties in adolescents (DuBois et al., 1992; Lindholdt et al., 2022). According to the systematic review from Bor et al. (2014), there has been in the 21st century an increase in the internalizing of problems (anxiety, depression), manifested mainly in girls, compared to the 20th century. Based on the meta-analytical results of Polanczyk et al. (2015), the prevalence of anxiety and depression among children and adolescents is 6.5% and 2.6%, respectively. In times of pandemic, there is even more risk of mental health problems, manifested in symptoms of depression, anxiety, or posttraumatic stress disorder (PTSD) (e.g., Douglas et al., 2009; Liu et al., 2020). According to a meta-analysis across 29 samples and 80,879 youth, the pooled prevalence of clinically elevated depression and anxiety symptoms during COVID-19 was 25.2% and 20.5%, respectively (Racine et al., 2021). According to a systematic review by Loades et al. (2020), there is a clear association between social isolation, loneliness and mental health problems arising during the pandemic among children and adolescents. Because socialization is very important for people in this age, the restrictions related to the pandemic may also play a significant role in mental health difficulties (e.g., Cohen et al., 2021). Adolescents with psychiatric history are an even more vulnerable group, possibly due to the disruption of psychiatric/psychological care, higher COVID-19-related anxiety, and possible difficulties in coping with lockdowns (e.g., Guessoum et al., 2020). Conversely, resilience, as an ability to cope with or bounce back from stressful events, may act as a protective factor against negative mental health outcomes (e.g., Hu et al., 2015; Anyan and Hjemdal, 2016; Li and Miller, 2017). According to Beames et al. (2021), higher resilience is related to decreased psychological distress and to increased positive outcomes. Their research pointed out that the most reported active coping strategies among Australian adolescent were socializing, engaging in hobbies, and exercise, which also highlight the importance of peer relationships for teenagers. This research also describes gender as an important factor related to resilience, where female gender was related to lower resilience. When comparing students based on age, younger students experienced significantly more distress associated with COVID-19 (e.g., Zhou et al., 2020).

Another important factor influencing the mental health of adolescents is attachment. While insecure attachment is linked with the development of internalizing problems, anxiety, and depression in adolescents (Brumariu and Kerns, 2010), more secure attachment to parents is associated with fewer depressive symptoms in this group (Kerstis et al., 2018). Attachment theorists emphasize the importance of sensitive and responsive interactions between parent and child for building trust towards oneself and others and for learning self-regulation strategies (Bowlby, 1969). The quality of early caregiving experiences and attachment style may affect stress responsivity: Securely attached adolescents may be more successful in self-regulating strategies and in adaptive coping responses (Howard and Medway, 2004). Anxiously (preoccupied) attached adolescents tend to engage in emotion-focused coping, such as rumination and self-blame, and to focus their attention on their own distress rather than focusing on solutions to current problems (Mikulincer et al., 2003). Avoidantly attached adolescents attempt to deal with distress and threats alone, using suppression and self-reliant strategies (Brenning et al., 2012). While relationships with caregivers/parents usually consist of the primary attachment bond, in adolescence, peer relationships begin to increasingly take on critical attachment functions (Allen and Tan, 2016).

Pandemics may be a challenging stressor that activates the attachment system. Individuals with higher attachment anxiety may be prone to experience more health anxiety, leading to maladaptive responses to the COVID outbreak (e.g., excessive hand washing, social isolation, extended fear from infection) (Asmundson and Taylor, 2020). In an Italian population sample, features of both secure and avoidant attachment style appeared to be protective for the risk of higher psychological burden during the COVID-19 outbreak compared to the anxious attachment style (e.g., Moccia et al., 2020).

Although a systematic review of longitudinal cohort studies showed that after an initial increase in mental health symptoms after the outbreak of COVID-19, there was decrease of problems comparable to pre-pandemic levels in mid-2020 among most population subgroups (e.g., Robinson et al., 2022). From a mental health prevention perspective, it may be important to also focus on younger age groups. Adolescents may be a particularly vulnerable group, as biopsychosocial stressors can have far-reaching consequences for future mental health, due to neuro-immuno-endocrinological changes induced by the stress of social isolation (de Figueiredo et al., 2021).

Based on the mentioned facts about potential risk and protective factors influencing psychological adjustment to the pandemic, the main goal of present paper was to examine COVID-19 anxiety and its association with sociodemographic characteristics, mental and physical health, resilience, attachment anxiety and avoidance among adolescents in Slovakia. We hypothesized that (1) girls will have higher COVID anxiety; (2) impaired mental and physical health will be associated with higher COVID anxiety; (3) adolescents with higher attachment anxiety will have higher score in COVID anxiety; and (4) less resilient adolescents will have higher score in COVID anxiety.

## Materials and methods

### Participants and data collection

Data was collected online from 13 April to 24 May 2021. In Slovakia, the state of emergency lasted from 1 October 2020 until 14 May 2021. Schools were in the distance learning regime, except the resumed full-time learning for the first class of primary and the last class of secondary school from 8 February 2021. Data from a total of 1786 adolescents were collected (age 15–19 years, mean age = 16.8, SD = 1.2). Two-thirds (66.3%) of the research sample were females (*N* = 1,184) and all the respondents were high-school students. The participants received a web link for the survey from their school management. Data collection was in line with the Declaration of Helsinki, and participation in the study was voluntary and anonymous. No incentives were offered for taking part in the survey. The study was reviewed and approved by the Scientific Ethics Committee of Palacky University Olomouc (NO 2021/11).

### Measures

**Demographics—**participants were asked to report their gender (male, female), age (continuous) and type of school where they were studying.

### COVID anxiety scale

The COVID Anxiety Scale (Silva et al., 2020) contains 7 items that can be answered on a 4-point Likert scale, where 0 = not applicable to me, 3 = very applicable to me. The reliability of the scale is *ω*_total_ = 0.92 (mean score = 6.25, SD = 5).

### SF-8 health survey

The SF-8 Health Survey consists of 8 items, each of them representing a specific domain: (1) general health (GH), (2) physical functioning (PF), (3) role physical (RP), (4) bodily pain (BP), (5) vitality (VT), (6) social functioning (SF), (7) mental health (MH), and (8) role emotional (RE). The Slovak version of the questionnaire is similar to the Czech version (Bartuskova et al., 2018), where the participant is asked to evaluate subjective health in the last 4 weeks. Items 1–4 can be answered on a 6-point scale and items 5–8 on a 5-point scale. In the presented study, we worked only with item 1 (general health) and item 7 (mental health). For the purpose of this study, the scoring of these items was reversed so that a higher score corresponds to better health.

### Brief resilience scale

The Brief resilience scale (BRS, Smith et al., 2008) consists of 6 items that can be answered on a 5-point Likert scale (1 = strongly disagree, to 5 = strongly agree). The BRS measures resilience as the ability to recover from a stressful experience. The Slovak version of the BRS shows good psychometric properties, reliability and validity (Furstova et al., 2021). The reliability of the scale on the research sample was *ω*_total_ = 0.86 (mean score = 2.98, SD = 0.9).

### The experiences in close relationships-revised for adolescents

The ECR-R questionnaire for adolescents is a self-report measure consisting of 20 items, with 10 items representing (1) attachment anxiety and (2) attachment avoidance (Wilkinson, 2011). The items can be answered on a 7-point Likert scale (1 = strongly disagree, to 7 = strongly agree). The reliability of the attachment anxiety subscale was *ω*_total_ = 0.89 (mean score = 37.08, SD = 14.11), and of the attachment avoidance it was *ω*_total_ = 0.80 (mean score = 29.93, SD = 10.73).

### Statistical analyses

First, the reliabilities of the measures were calculated using the total omega coefficient. For the main analysis, several nested linear regression models were estimated. The dependent variable was COVID anxiety, while gender, age, resilience and attachment anxiety and attachment avoidance were treated as predictors. The psychological variables were standardized. The predictors were divided into 3 blocks [(1) sociodemographics – age, gender, (2) sociodemographics, resilience, (3) sociodemographics, resilience, attachment avoidance, and (4) sociodemographics, resilience, attachment avoidance, and attachment anxiety]. Each one of the 4 blocks is presented as an individual model (see Table 1). The smallest effect size of interest (SESOI) for these models was set to *β* = 0.10 (based on Cohen, 1988).

**Table 1:** Results of linear regression models assessing the effect of background characteristics, health (SF-8), resilience (BRS) and attachment anxiety and avoidance (ECR-R) on the level of CAS.

Predictor	Model 1		Model 2		Model 3		Model 4	
	Beta	Std. error	Beta	Std. error	Beta	Std. error	Beta	Std. error
Background characteristics								
Gender (male vs. female)	−0.50***	0.05	−0.31***	0.05	−0.26***	0.05	−0.26***	0.05
Age (years)	0.05*	0.02	0.05*	0.02	0.05**	0.02	0.06**	0.02
School								
Art school (conservatory)	reference							
Grammar school	−0.36*	0.16	−0.27	0.15	−0.25	0.15	−0.25	0.15
Vocational school	−0.36*	0.15	−0.26	0.15	−0.25	0.15	−0.26	0.15
Apprenticeship	−0.36*	0.16	−0.27	0.16	−0.27	0.15	−0.28	0.15
Health (SF-8)								
General health			−0.14***	0.03	−0.12***	0.03	−0.13***	0.03
Mental health			−0.20***	0.03	−0.15***	0.03	−0.14***	0.03
Resilience (BRS)					−0.14***	0.03	−0.12***	0.03
Attachment (ECR-R)								
Anxiety							0.09***	0.03
Avoidance							−0.11***	0.02
*R* ^2^	0.061		0.140		0.154		0.167	
*R*^2^ difference			0.079		0.014		0.013	

*p < 0.05; **p < 0.01; ***p < 0.001.

Afterwards, the Pearson correlation coefficient was calculated to examine the relationship between general health, mental health (variables measured by the SF-8 Health Survey) and COVID anxiety level. All the statistical analyses were performed in the R software, version 4.6.1 (R Core Team, 2020).

## Results

### Background characteristics

The background characteristics of the Slovak adolescent sample are presented in Table 2. The data comprised 1,786 participants with the mean age of 16.8 years; 66.3% were female. A significantly higher level of COVID anxiety was reported by girls [*t*(10.12) = *p* < 0.001, Cohen’s *d* = 0.51]. Students attending art schools (conservatories) also reported higher mean COVID anxiety than students attending other types of high school; however, the difference was not significant. There were no significant group differences found between the counties and size of municipality either. COVID anxiety was positively correlated significantly with attachment anxiety and significantly negatively correlated with general health, mental health, and resilience (see Table 3).

**Table 2:** Descriptive characteristics of the data, comparison of COVID anxiety between sociodemographic groups.

Characteristic	*n* (%)	COVID anxiety scale (CAS)	
		Mean (SD)	Value of *p*
Total	1,786 (100)	6.23 (4.98)	
Gender			<0.001
Female	1,184 (66.3)	7.05 (5.03)	
Male	602 (33.7)	4.60 (4.46)	
School			0.082^a^
Art school (conservatory)	42 (2.4)	8.17 (5.69)	
Grammar school	406 (22.7)	6.26 (5.05)	
Vocational school	1,057 (59.2)	6.15 (4.90)	
Apprenticeship	281 (15.7)	6.15 (5.03)	
County			0.024^a^
Trenciansky	530 (29.7)	5.87 (5.00)	
Zilinsky	153 (8.6)	5.51 (4.25)	
Presovsky	372 (20.8)	6.93 (5.25)	
Banskobystricky	158 (8.8)	6.59 (4.81)	
Kosicky	204 (11.4)	6.38 (5.11)	
Bratislavsky	258 (14.4)	6.19 (4.98)	
Trnavsky	64 (3.6)	6.27 (5.11)	
Nitriansky	47 (2.6)	5.21 (4.09)	
Municipality size			0.55
Less than 1,000	290 (16.2)	6.03 (5.13)	
1,000 to 2,000	246 (13.8)	6.07 (4.57)	
2,000 to 5,000	319 (17.9)	6.54 (5.12)	
5,000 to 20,000	351 (19.7)	6.05 (4.89)	
20,000 to 50,000	229 (12.8)	6.21 (4.85)	
50,000 to 100,000	233 (13.0)	6.11 (5.06)	
More than 100,000	118 (6.6)	6.95 (5.41)	

Comparison of groups was performed with *t*-test and one way ANOVA.

a There were no significant group differences found with the post-hoc Sheffe test.

**Table 3:** Correlation coefficients between the CAS, age, health (SF-8), resilience (BRS), and attachment anxiety and avoidance (ECR-R).

	Mean (SD)	Correlation coefficient with CAS	Value of *p*
Age	16.79 (1.15)	0.05	0.058
Health			
SF-8 General health	4.28 (1.23)	−0.27	<0.001
SF-8 Mental health	3.10 (1.30)	−0.32	<0.001
Resilience			
BRS sum score	2.97 (0.90)	−0.29	<0.001
Attachment			
ECR-R anxiety subscale	37.15 (14.10)	0.27	<0.001
ECR-R avoidance subscale	39.91 (10.70)	0.04	0.063

CAS, COVID Anxiety Scale; Pearson correlation coefficients are reported.

### Predictors of adolescent COVID anxiety

The effect of the background characteristics of the participants, health, resilience and attachment anxiety and avoidance on the level of adolescent COVID anxiety was assessed using nested linear regression models. In Model 1, the background characteristics explained 6.1% of the total variance of the COVID anxiety. In this model, a higher level of the COVID anxiety was associated with female gender, higher age and students attending art schools (see Table 1). After adding health to the model (Model 2), the proportion of the explained variance increased by 7.9%. The effect of general and mental health was significant; a higher score in the health domains was associated with a lower level of COVID anxiety. In Model 3, after adding resilience to the model, the explained variance increased by another 1.4%. Resilience was found to significantly decrease the level of COVID anxiety. In Model 4, attachment anxiety and avoidance were added. Attachment anxiety increased the level of COVID anxiety, while attachment avoidance had a decreasing effect. The final explained variance reached 16.7%. In Models 2, 3, and 4, the type of school lost its significance. In the final model, the significant predictors with an acceptable effect size (above the smallest effect size of interest, SESOI, *β* > 0.10) were all associated with a decreased level of COVID anxiety: male gender, higher general and mental health score, and higher resilience and attachment avoidance scores.

## Discussion

The main goal of this paper was to explore the relationships between COVID anxiety and its risk/protective factors. Negative predictors (protective factors) of COVID anxiety were being male, higher resilience and attachment avoidance. On the other hand, higher age, worsened general and mental health, and attachment anxiety were risk factors for COVID anxiety. The effect of other variables in the model were negligible (i.e., bellow our SESOI).

### The effect of background characteristics

Mental health problems are in general more prevalent in females than in males (e.g., McLean et al., 2011; Baxter et al., 2014; Salk et al., 2017). Females are more vulnerable to developing psychological symptoms, such as anxiety, after a stressful or traumatic event (Tolin and Foa, 2008). The present findings corroborate girls experiencing higher level of COVID anxiety (e.g., Racine et al., 2021; Evren et al., 2022; Mora-Magaña et al., 2022). We also found that adolescents are more likely to be COVID anxious with increasing age. This is in line with other research that has observed higher mental health symptomatology in outcomes such as anxiety or depression in older adolescents (e.g., Nearchou et al., 2020). Regarding the type of school, higher mental health outcomes were observed in music and art students (e.g., Spahn et al., 2004; Vaag et al., 2021). Based on this fact, we used art school students as a reference group when comparing students in COVID anxiety levels; however, this difference was not significant.

### The effect of health

In the context of the pandemic, much research has been done on both mental and physical health (e.g., Lakhan et al., 2020; Cui et al., 2022). Research findings based on longitudinal data have typically suggested that people’s mental and physical health worsened during the pandemic. Nonetheless, pre-pandemic levels of mental health should also be taken into consideration (e.g., Cui et al., 2022). Several studies have confirmed that these baseline levels were a strong risk factor for mental health difficulties during the pandemic (Czeisler et al., 2020; McGinty et al., 2020; Fancourt et al., 2021; Shanahan et al., 2022). For instance, people who reported higher levels of anxiety even before the pandemic experienced a steeper decline in mental health during the pandemic (e.g., Morales et al., 2022). Even though we do not have the pre-pandemic data, our results suggest that general/mental health is a significant predictor of COVID anxiety. This is debatable, however, as general health could be both the cause and a consequence of perceived COVID related anxiety in adolescents.

### The effect of resilience

Resilience as the ability to bounce back from a stressful event is very important in coping with stress (Rutter, 2018), especially in pandemic times (e.g., Seaborn et al., 2022). In general, resilience plays a significant role in mental health. Based on current research, it might be a protective factor for anxiety related specifically to COVID-19 (e.g., Barzilay et al., 2020; Skalski et al., 2021), as well as general anxiety, which also applies during times of pandemic (e.g., Taao et al., 2022). Besides the importance of resilience during stressful times, it is also a relevant predictor of subsequent mental health. According to a Chinese study, a higher level of resilience before COVID-19 significantly predicted a decreased level of depression and anxiety after periods of lockdown (Shi et al., 2022). The protective model of resilience was supported by various researchers, who pointed out some factors that are specifically related to better mental health outcomes. According to Askeland et al. (2020), greater goal orientation, self-confidence, social competence, social support and family cohesion are important for maintaining mental health in adolescents. Similarly, a study on the risk and resilience of adolescents during COVID-19 showed that the most robust associations with teens’ distress were with feelings of stress around parents and support received from them (Luthar et al., 2021). Similar results were obtained in a meta-analysis focusing on social support and its role in anxiety and other mental health variables in childhood and adolescence (Rueger et al., 2016; Heerde and Hemphill, 2018). Altogether the data suggest that interventions should attend not just to adolescents’ mental health but also that of caregiving adults at home and school. There are strong associations between adolescents’ reports of prosocial and health-protective behaviors, as well as significant pathways to COVID-19 prosocial health protective behaviors from parent–adolescent attachment security through adolescents’ favorable mental health responses to the pandemic (Coulombe and Yates, 2022). Therefore, interventions aimed at boosting these potential protective factors would be beneficial for adolescents (not only) in pandemic times. Promote the well-being of adolescents (and others) during this difficult crisis should consider the quality of parent–adolescent relationships with fostering adolescent’s felt security and safety with attachment-based interventions (review in Mikulincer and Shaver, 2007).

### The effect of attachment anxiety and attachment avoidance

Based on our results, attachment anxiety is positively correlated to COVID anxiety. This result is in line with an Italian study, where the relationship between various attachment styles (secure attachment, fearful attachment, dismissing attachment and preoccupied attachment) and COVID anxiety was explored. Fearful and preoccupied attachment were significant positive predictors of COVID anxiety. This could be explained by the fact that people with higher attachment anxiety may tend to be more preoccupied about the pandemic and its related restrictions, which could lead to higher anxiety related to the COVID. In accordance with other research findings, attachment avoidance in our study was negatively correlated with the COVID anxiety (e.g., Moccia et al., 2020; Vismara et al., 2022). This relationship may be explained by the fact that avoidantly attached individuals, who tend to be self-directed and may exhibit less distress in social isolation, may have perceived the pandemic and restrictions as less stressful (in comparison with anxiously attached individuals). On the other hand, even in avoidantly attached people, social distancing and possible long-lasting loneliness related to pandemic restrictions might have an impact on mental health (e.g., Vismara et al., 2022). Based on available studies showing associations between attachment and the psychological impact of the pandemic (e.g., Moccia et al., 2020; Coulombe and Yates, 2022; Vismara et al., 2022), we can assume that secure attachment may play a key role in protection before the emergence of mental health disorders in challenging pandemics times. Securely attached people face stressful events relying on both others’ support and their own self-confidence; they have the capacity to mitigate loneliness, which reduces potential anxiety and mental health problems.

Adolescence is an especially sensitive period for brain development (Fuhrmann et al., 2015). Bio-psycho-social stressors related to the pandemic may have an impact on mental health due to neuro-immuno-endocrinological changes induces by stress (de Figueiredo et al., 2021). Since socialization and relationships are in general very important for people in this age, the social isolation and potential long-term feeling of loneliness may be important sources of distress. The rapid systematic review of studies from various pandemics (Loades et al., 2020) showed that the length of loneliness due to pandemic restrictions in particular appears to be a predictor for future mental health. This could be taken into consideration when planning pandemic restriction rules for schools, and children and adolescents should be prevented from taking part in long periods of social distancing.

Based on fact that in adolescence peer relationships begin to increasingly take on critical attachment functions (Allen and Tan, 2016) and the security in peer attachment relationships is related to youths’ feeling of connection (Parent et al., 2021), it is also important to focus on peer attachment relationships, because they can serve as an important source of social support, intimacy and strength and serve as a protective factor in situations of chronic stress during a pandemic. Simultaneously, it is important to aim to improve resilience, for example, by using a mindfulness training program (Yuan, 2021) or promoting active coping skills.

### Limits and perspectives for future research

The present study has several limitations. The first regards the collected variables. The participants were not asked about any psychiatric diagnosis in their history or their mental health difficulties before the pandemic. They were also not asked if they are/were positive for COVID-19, which could have potentially affected their mental health. In both cases, the information could have helped us describe the anxiety related to COVID-19 more properly. Because our study design is cross sectional and we do not have pre-pandemic data from the same population, we cannot explore causality.

## Conclusion

The pandemic had a significant impact on various domains of life, including mental health. Our study aimed to describe anxiety specifically related to COVID-19 and its association among Slovak adolescents. Based on our results, lower resilience, higher attachment anxiety, being a girl and having a higher age are predictors related to higher COVID anxiety. As resilience is a dynamic process and not a stable trait, interventions aimed at boosting resilience and preventing loneliness and social disconnection might have significant meaning when working with adolescents (not only) in pandemic times. Moreover, fostering secure attachment with parents and peers might promote prosocial behavior and the mental health of adolescents.

## Data availability statement

The raw data supporting the conclusions of this article will be made available by the authors, without undue reservation.

## Ethics statement

The studies involving human participants were reviewed and approved by Scientific Ethics Committee of Palacky University Olomouc. Written informed consent from the participants’ legal guardian/next of kin was not required to participate in this study in accordance with the national legislation and the institutional requirements.

## Author contributions

The authors collectively conceived the main idea and design of the study. BJ and NK wrote the theoretical framework and discussion, with input from JH, GS, and JF. BJ performed the statistical analyses, with input from JF. JH, NK, and PT supervised the study. All authors contributed to the article and approved the submitted version.

## Funding

This study was supported by Palacky University Olomouc (project no. DSGC-2021-0122) and the Slovak Psychiatric Association (project no. 01/2021).

## Conflict of interest

The authors declare that the research was conducted in the absence of any commercial or financial relationships that could be construed as a potential conflict of interest.

The reviewer FS declared a shared affiliation with the authors NK and GS to the handling editor at the time of review.

## Publisher’s note

All claims expressed in this article are solely those of the authors and do not necessarily represent those of their affiliated organizations, or those of the publisher, the editors and the reviewers. Any product that may be evaluated in this article, or claim that may be made by its manufacturer, is not guaranteed or endorsed by the publisher.
